# Investigation of Susceptibility Levels of *Culex pipiens* L. (Diptera: Culicidae) Populations to Synthetic Pyrethroids in Antalya Province of Turkey

**Published:** 2019-09-30

**Authors:** Onder Ser, Huseyin Cetin

**Affiliations:** 1 Malaria Control Unit, Antalya Provincial Directorate of Health, Antalya, Turkey; 2 Department of Biology, Faculty of Science, Akdeniz University, Antalya, Turkey

**Keywords:** Antalya, *Culex pipiens*, Mosquito, Resistance, Synthetic pyrethroid

## Abstract

**Background:**

*Culex pipiens* L. (Diptera: Culicidae) is an important vector of several pathogens. This mosquito is widely distributed throughout the world. We aimed to determine the susceptibility levels of *Cx*. *pipiens* populations to some synthetic pyrethroid insecticides in Antalya, Turkey.

**Methods:**

The immature stages of mosquitoes were collected from eight locations in Alanya, Döşemealtı, Kemer, Kumluca, and Manavgat districts of Antalya between Apr and Oct of 2017. Adult susceptibility tests were carried out according to a modified version of the Centers for Disease Control and Prevention bottle bioassay. In the tests, the World Health Organization recommended diagnostic doses; permethrin (0.75%), etofenprox (0.5%), deltamethrin (0.05%) and lambda-cyhalothrin (0.05%) were used.

**Results:**

As a result of the susceptibility tests, deltamethrin was the least effective insecticide and it caused 58.78–97.56% mortalities on *Cx. pipiens* populations while permethrin was the most effective substance that caused 100% mortality on all populations. While all of the tested populations were found susceptible to permethrin, and possible resistant or resistant to deltamethrin. Etofenprox and lambda-cyhalothrin led to 91.54–100% and 93.1–100% mortalities, respectively.

**Conclusion:**

The possible resistance or resistance to deltamethrin in all the areas is caused by the widespread use of this chemical against pests in agriculture and public health applications for long-term. Moreover, a concordance was found between resistance levels and the intensity of pesticide application in agriculture and public health, and organic and chemical pollution levels in the sampled habitats.

## Introduction

There are over 3500 species of mosquitoes (Diptera: Culicidae) in the world, and more than 50 of them have been documented in Turkey (1, 2). *Culex pipiens* L. is an important vector of several disease-causing pathogens, such as filarial nematode (*Wuchereria bancrofti*), West Nile virus (WNV), Rift Valley fever virus, and St. Louis encephalitis virus. This mosquito species is widely distributed throughout the world (3, 4). *Culex pipiens* is the predominant species or it is intensively encountered in mosquito fauna studies conducted in different provinces of Turkey (1, 5–8). *Culex pipiens* was also seen as a dominant mosquito species in studies carried out in Antalya Province (9, 10). This mosquito is the possible vector of the WNV in Turkey (11, 12). In 2010, 47 WNV infections were detected and 10 patients died from the WNV infection ( 12 ).

One of the most effective manners of controlling mosquito-borne diseases transmission is to control of their vectors. Despite the use of various methods to control mosquitoes; the application of insecticides continue to be the most preferred method due to easy accessibility, fast and effective results in a short time. However, excessive and unconscious use of insecticides lead to various problems in terms of environment and human health and also cause to development of resistance by mosquitoes. Parallel to resistance development, control of mosquito populations are becoming more difficult and it may be an increase in the incidence of mosquito-borne diseases (13–15). Therefore, it is essential to monitor the insecticide resistance and susceptibility in field mosquito populations to ensure the sustainability of mosquito control programs (4).

Synthetic pyrethroids (SP) were produced to increase the chemical stability and biological activity of natural pyrethrins, which have insecticidal effects. Natural pyrethrins are obtained from the extraction of dried flower heads of *Chrysanthemum* spp. These contain a mixture of insecticidal action esters. SP are neurotoxic effect to insects and their primary action site is the voltage-gated sodium channels (16, 17). The mode of action of these insecticides is similar to the organic chlorine insecticide, dichloro diphenyl trichloroethane (DDT). These insecticides act the voltage-gated sodium channels on the membranes of the nerve cells and cause over-stimulation due to the longer opening of channels (18, 19). Traditionally, SP are classified into two groups namely type I and type II, according to their chemical structure and toxicology. Type I pyrethroids do not contain the α-cyano group, whereas type II pyrethroids include the α-cyano group on the phenoxybenzyl moiety (19). SP have killer, knock-down and repellent effects on insects. In addition, these insecticides can be used with synergistic compounds to increase their activity (16). SP have several advantages, as compared with other insecticides in terms of cost, safety (less toxic to mammals), repellency, and duration of residual action (20). SP are broad-spectrum insecticides, effective against a number of insect pests (21). Currently, these insecticides are widely used, in agriculture, public health, veterinary medicine and as household pesticides (20, 21). Using them as larvicides are limited due to their toxicity against non-target aquatic organisms. Nowadays, SP are used on all certified long-lasting insecticidal nets and indoor residual spraying programmes for control of major vector-borne diseases worldwide (20).

We aimed to determine the susceptibility levels of *Cx. pipiens* populations collected from different districts of Antalya, important tourism and agricultural center of Turkey, to some synthetic pyrethroid insecticides commonly used against mosquito adults.

## Materials and Methods

### Mosquitoes

**Fig. 1.**
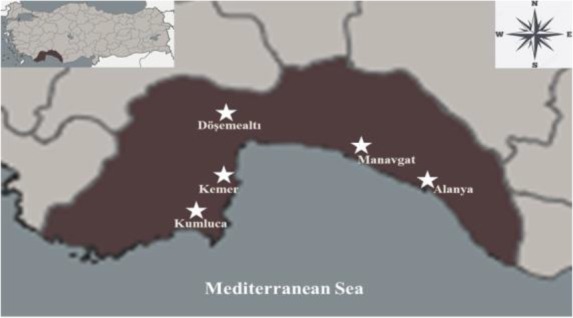
*Culex pipiens* collection sites in various districts of Antalya Province, Turkey in 2017

The immature stages (egg raft, larva, and pupa) of mosquitoes were collected from aquatic habitats in Alanya (Çıplaklı and Süleymanlar), Döşemealtı (Ilıca and Killik), Kemer (Tekirova), Kumluca (Naranciye and solid waste storage area) and Manavgat (Çakış) districts of Antalya between Apr and Oct of 2017 (Fig. 1). Global positioning system (GPS) coordinates of the sampling areas are shown in Table 1. The immature stages collected from breeding sites were transported to the Department of Biology, Faculty of Science, Akdeniz University and reared to adults under standard conditions (at a temperature of 25±2 °C, 60±10% relative humidity and 12h light: 12h dark photoperiod in an insectary). Larval feeding was done by using fish food. A pad of cotton soaked in 10% sucruse solution was provided for adult mosquitoes feeding. The species identification was made using the morphological characters according to the identification keys (16, 22, 23).

**Table 1.** Location and global positioning system (GPS) coordinates of *Cx. pipiens* collection sites

**No**	**District**	**Location**	**GPS Coordinate**
1	Alanya	Çıplaklı	36º33′39.9″ N 32º02′44.2″ E
2	Alanya	Süleymanlar	36º40′42.299″ N 31º59′04.256″ E
3	Döşemealtı	Ilıca	37º09′12.868″ N 30º37′54.912″ E
4	Döşemealtı	Killik	37º12′51.987″ N 30º39′52.588″ E
5	Kemer	Tekirova	36º31′04.634″ N 30º32′18.758″ E
6	Kumluca	Narenciye	36º21′59.7″ N 30º17′21.3″ E
7	Kumluca	Solid waste storage area	36º25′10.538″ N 30º18′30.969″ E
8	Manavgat	Çakış	36º54′46.150″ N 31º09′47.825″ E

### Insecticides

In the susceptibility tests, synthetic pyrethroid active substances (permethrin, etofenprox, deltamethrin, and lambda-cyhalothrin) were purchased from Tagros Chem. India Ltd. and used at the diagnostic doses recommended by the World Health Organization (WHO) (20).

### Bioassays

Adult susceptibility tests were carried out according to a modified version of the Centers for Disease Control and Prevention (CDC) bottle bioassay (24). The WHO susceptibility assay and the CDC bottle test are the most frequently used methods for detection of insecticide resistance in mosquito populations (25). The results obtained from WHO and CDC test methods support each other. Similar results were found between the two methods in terms of mortality rates in the studies conducted to determine the susceptibility levels to insecticides in malaria vectors (26, 27). Both methods have several advantages and disadvantages. For example, WHO test kits are expensive and may not be easily attainable, but CDC bottle test method is simple, fast and cost-effective (25–27). However, the licensing of insecticides used in indoor residual spraying applications in Turkey is carried out according to doses recommended by the WHO. In addition, the doses used in the CDC bottle test method are considerably lower than the doses used in the WHO susceptibility tests. Therefore, the diagnostic doses and time recommended in the WHO (2013) test procedures (20) were used in our study. For this purpose, WHO recommended stock solutions (0.75% permethrin, 0.5% etofenprox, 0.05% deltamethrin and 0.05% lambda-cyhalothrin) were prepared by dissolving the active substances in acetone. For each synthetic pyretroid, 1.836ml of stock solution was applied to the inner surface of the glass jars having an inner surface area of about 500cm^2^ and solution spreads in the jar. In this way, an equal amount of the active substance to the amount of per square centimeter insecticide on the WHO tube test method papers was applied to the glass surfaces (20, 25, 28). After the solvent was evaporated (2h waiting period), tests were performed with 20–40 non-blood fed, 3–5 day-old adult female mosquitoes. These individuals were recorded with knock-down rates at 5min intervals for 1h.

After the 1h exposure period, mosquitoes were transferred to clean jars for recovery. The number of dead mosquitoes in both the treated and the control jars was recorded 24h post-exposure. According to the WHO recommendations, mosquitoes are categorized as dead if they are immobile or unable to stand on or fly in a coordinated manner. Each bioassay was conducted at least using four replicates. In each bioassay, one control group was used. Only acetone is applied to the jars where the control groups are located and the inside surfaces are dried.

### Statistical analysis

Corrected mortality rate was calculated using Abbott's formula when mortality rate in the control group was between 5–20% (29). Values of times for 50% knockdown (KDT_50_) and KDT_95_ were calculated by Stat-Plus probit analysis program. Insecticide resistance status of populations was assessed according to WHO (2013) (20) criteria, where mortality range 98–100% was susceptible, 90–97% possible resistant, and < 90% resistant.

## Results

### Knock-down effect of synthetic pyrethroids

The KDT_50_ and KDT_95_ values of active substances are presented in Table 2. At the end of 1h, while the knock-down rates for permethrin were 100% in all populations, the knock-down rates for etofenprox, deltamethrin and lambda-cyhalothrin were between 88.21–100%, 31.31–99.58% and 94.27–100%, respectively. According to KDT_50_ values, permethrin showed the highest knock-down effect on *Cx. pipiens*, except for the Döşemealtı-Ilıca population. Although different results were obtained for other active substances, deltamethrin showed the highest KDT_50_ values and the lowest knockdown effect at the five populations. KDT_50_ values of permethrin were 6.00–12.10min, of etofenprox, were 6.57–27.61 min, of deltamethrin were 11.26–122.53min and of lambda-cyhalothrin were 5.57–20.66 min. The lowest KDT_50_ value in all populations was 5.57min at lambda-cyhalothrin in the Döşemealtı-Ilıca population, while the highest KDT_50_ value was 122.53min at deltamethrin in the Kumluca-solid waste storage area population. When KDT_95_ values were compared, deltamethrin showed the lowest knock-down effect except for the Döşemealtı-Killik population while permethrin showed the highest knock-down effect on all populations. KDT_95_ values of permethrin were 12.96–29.59 min, of etofenprox were 17.36–85.36min, of deltamethrin were 40.83–744.30min and of lambda-cyhalothrin were 14.82–69.66min in all populations. The lowest KDT_95_ value of all populations was 12.96min at the permethrin in the Döşemealtı-Ilıca population, while the highest KDT_95_ value was 744.30min at the deltamethrin in the Kumluca-solid waste storage area population.

**Table 2.** Knock-down (KD) rates, mortality rates, KDT_50_ and KDT_95_ values and susceptibility status of *Cx. pipiens* populations from Antalya, Turkey to four synthetic pyrethroids

**Populations**	**Parameters**	**Permethrin (0.75%)**	**Etofenprox (0.05%)**	**Deltamethrin (0.05%)**	**Lambda-cyhalothrin (0.05%)**	**Control**
**Alanya-Çıplaklı**	N	165	172	190	167	171
	%KD±SE (after 60min)	100	88.21±5.40	80.03±5.09	94.27±1.47	0
	KDT_50_±SE (min)	12.10±0.82	26.02±0.82	26.85±1.24	20.66±0.73	
	KDT_95_±SE (min)	27.27±2.32	85.36±5.74	188.76±27.69	69.66±4.25	
	χ^2^	24.4440	2.0809	1.0577	3.1637	
	P-level	0.0065	0.9957	0.9998	0.9773	
	%Mortality±SE (after 24h)	100	91.54± 2.51	80.30±3.01	93.10±2.08	0.63±0.63
	Susceptibility status	S	PR	R	PR	
**Alanya-Süleymanlar**	N	115	112	131	121	102
	%KD±SE (after 60min)	100	94.34±2.67	92.96±4.32	100	0
	KDT_50_±SE (min)	11.81±0.68	27.61±1.43	22.89±0.82	14.79±0.48	
	KDT_95_±SE (min)	29.59±2.04	81.18±9.09	86.54±6.32	33.66±1.39	
	χ^2^	12.6888	17.5235	5.6160	4.5321	
	P-level	0.2416	0.0636	0.8464	0.9202	
	%Mortality±SE (after 24h)	100	91.95±3.37	90.23±2.45	99.19±0.81	0.96±0.96
	Susceptibility status	S	PR	PR	S	
**Döşemealtı-Ilıca**	N	118	110	124	138	114
	%KD±SE (after 60min)	100	100	65.98±7.32	100	0
	KDT_50_±SE (min)	6.00±0.30	6.57±0.37	47.19±1.41	5.57±0.36	
	KDT_95_±SE (min)	12.96±0.84	17.36±1.06	124.14±10.94	14.82±1.00	
	χ^2^	0.0344	0.0390	11.7727	0.0646	
	P-level	0.9999	0.9999	0.3006	0.9999	
	%Mortality±SE (after 24h)	100	100	76.51±4.92	100	5.15±3.16
	Susceptibility status	S	S	R	S	
**Döşemealtı-Killik**	N	158	154	196	169	144
	%KD±SE (after 60min)	100	98.33±1.67	99.58±0.42	100	0
	KDT_50_±SE (min)	9.89±0.41	15.85±0.58	13.09±0.55	12.03±0.46	
	KDT_95_±SE (min)	24.78±1.23	46.07±2.25	40.83±2.10	30.61±1.41	
	χ^2^	4.7670	4.6889	1.7015	0.8662	
	P-level	0.9062	0.9110	0.9982	0.9999	
	%Mortality±SE (after 24h)	100	99.17±0.83	97.56±0.86	100	0
	Susceptibility status	S	S	PR	S	
**Kemer-Tekirova**	N	118	121	139	115	109
	%KD±SE (after 60min)	100	100	92.00±8.00	100	0
	KDT_50_±SE (min)	6.37±0.35	8.96±0.44	11.26±0.70	12.01±0.50	
	KDT_95_±SE (min)	15.95±1.00	25.89±1.39	60.06±4.72	34.32±1.68	
	χ^2^	0.2756	1.1492	0.9450	1.2361	
	P-level	0.9999	0.9997	0.9999	0.9995	
	%Mortality±SE (after 24h)	100	100	91.70±4.39	100	1.71±1.05
	Susceptibility status	S	S	PR	S	
**Kumluca-Narenciye**	N	149	147	171	155	148
	%KD±SE (after 60min)	100	99.28±0.72	93.10±3.01	96.14±1.76	0
	KDT_50_±SE (min)	11.35±0.43	16.84±0.75	19.69±0.70	14.67±0.58	
	KDT_95_±SE (min)	26.91±1.24	41.77±2.47	65.40±3.85	45.94±2.37	
	χ^2^	5.8699	11.9181	1.8928	2.4029	
	P-level	0.8261	0.2906	0.9971	0.9922	
	%Mortality±SE (after 24h)	100	98.55±1.45	90.07±2.86	96.94±1.08	0
	Susceptibility status	S	S	PR	PR	
**Kumluca-Solid waste storage area**	N	137	134	138	115	138
	%KD±SE (after 60min)	100	100	31.31±2.52	100	0
	KDT_50_±SE (min)	8.99±0.34	11.93±0.72	122.53±20.66	9.88±0.37	
	KDT_95_±SE (min)	18.51±0.92	20.67±1.78	744.30±310.93	20.81±0.99	
	χ^2^	2.3809	32.7119	2.9895	0.7357	
	P-level	0.9925	0.0003	0.9817	0.9999	
	%Mortality±SE (after 24h)	100	100	58.78±5.34	100	2.48±1.78
	Susceptibility status	S	S	R	S	
**Manavgat-Çakış**	N	137	120	133	114	119
	%KD±SE (after 60min)	100	100	84.38±4.61	100	0
	KDT_50_±SE (min)	8.96±0.39	12.40±0.48	24.82±0.87	13.85±0.47	
	KDT_95_±SE (min)	21.94±1.13	32.58±1.52	95.55±7.47	32.08±1.36	
	χ^2^	0.0921	1.8646	4.6029	7.4133	
	P-level	0.9999	0.9973	0.9161	0.6859	
	%Mortality±SE (after 24h)	100	100	80.67±5.76	100	7.93±4.78
	Susceptibility status	S	S	R	S	

N: Number of individuals tested, PR: Possible Resistant, R: Resistant, S: Susceptible, SE: Standard Error

### Mortality rates

**Fig. 2.**
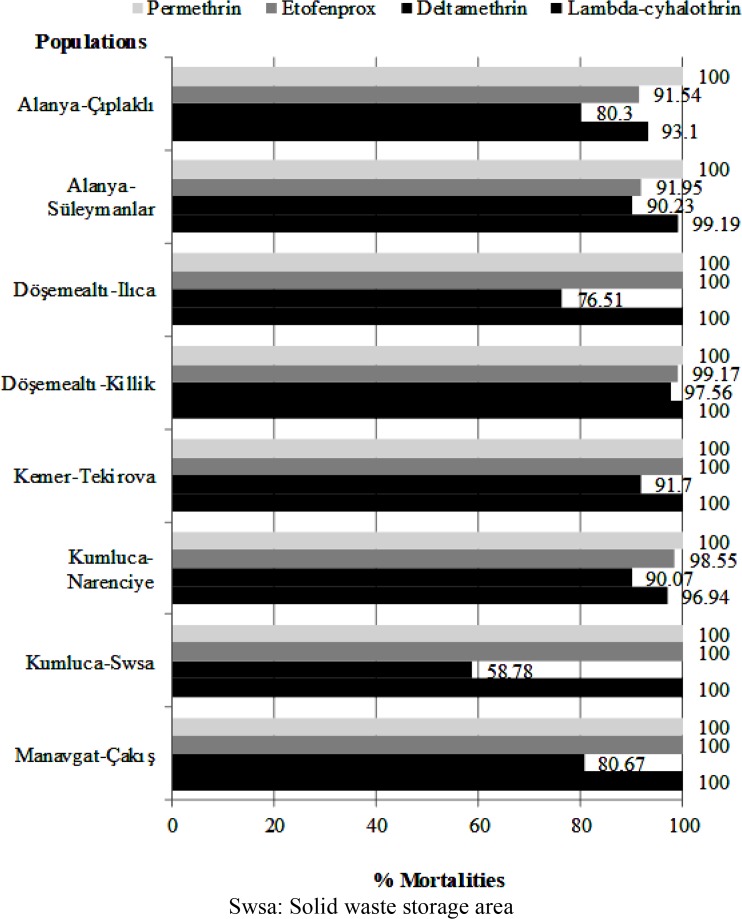
Mortality rates of *Cx. pipiens* populations to diagnostic concentrations of synthetic pyrethroids

As a result of the susceptibility tests, deltamethrin was the least effective insecticide and it caused 58.78–97.56% mortalities on *Cx. pipiens* while permethrin was the most effective substance that caused 100% mortality on all populations (Fig. 2). According to WHO criteria, while all of the tested populations were susceptible to permethrin, none of the populations were susceptible to deltamethrin, four of the eight populations were resistant to deltamethrin and the remains were possible resistant (Table 2). In addition, the lowest mortality rate among all tested populations was obtained from deltamethrin at 58.78% in the Kumluca-solid waste storage area population. Etofenprox and lambda-cyhalothrin led to 91.54–100% and 93.10–100% mortality in the eight tested populations, respectively (Fig. 2). Populations collected from two sampling sites for each of etofenprox and lambda-cyhalothrin were determined as possible resistant and the other six populations were susceptible (Table 2).

## Discussion

### Knock-down rates

SP are rapid-acting insecticides, which have a knock-down effect (20). The target site of these insecticides is voltage-gated sodium channels of nerve cell membranes in insect body (30, 31). Mutations in genes encoding the amino acid sequence in these channels cause a reduction in the sensitivity of the channels to the binding of pyrethroid insecticides. Alterations in the target site that lead to resistance to insecticides are usually referred to as knockdown resistance or kdr (32). In the case of kdr in a insect population, the rates of knock-down obtained from susceptibility tests are an important parameter for early detection of resistance to the insecticide (20). From this point of view, there is currently no resistance development against permethrin in all studied populations due to 100% knockdown rates at the end of 1h and low KDT_50_ and KDT_95_ values. Permethrin is a highly active substance with rapid knockdown effect against a variety of insects (33). However, intensive use of permethrin may cause development of resistance in mosquito populations. Extensive use of permethrin and DDT is involved in the selection of resistance against these insecticides in field populations of *Anopheles* (*An.*) *gambiae* in Burkina Faso (34). While mosquitoes collected from cotton-growing and urban areas were resistant to permethrin and DDT, mosquitoes collected from areas with limited insecticide selection pressure (rice fields and control areas) were susceptible. Mosquitoes in all tested areas were susceptible to deltamethrin with high mortality rates. In addition, mosquitoes collected from cotton-growing and urban areas showed higher KDT_50_ and KDT_95_ values with permethrin and DDT than deltamethrin. Resistance to permethrin and DDT may explain extensive use of these insecticides in the cotton-growing area and domestic use of same insecticides as bomb spray or coils in the urban areas.

The appearance of 31.31–99.58% knockdown rates at the end of 1h and high values of KDT_50_ (11.26–122.53min) and KDT_95_ (40.83–744.30min) of deltamethrin in this study indicate resistance development in all studied populations against this active substance. Susceptibility to deltamethrin of field-collected populations and colonized strains of *An. culicifacies* were investigated in India (35). Although there was 100% mortality in all the strains of *An. culicifacies* exposed to diagnostic concentration of deltamethrin for 1h, knockdown bioassays revealed more than two-fold higher values of KDT_50_ and KDT_90_ in *An. culicifacies* from Rameshwaram Island (both in field-collected and colonized strain), than populations from other areas. Results indicated the development of incipient resistance to deltamethrin in this strain of *An. culicifacies*. Insecticide resistance levels were evaluated in nine populations of *An. gambiae* sampled in three areas in the east of Tanzania (36). These areas are represented as an agriculture area, an urban area and a low pollution area depending on urbanization, agriculture activity, and usage of insecticides for vector control. For adult mosquitoes, resistance ratio fifty (RR_50_) was obtained by dividing the KDT_50_ of each population to the KDT_50_ of the susceptible reference strain. Adult susceptibility tests revealed that populations from urban and agriculture areas demonstrated the moderate resistance levels to deltamethrin with mean RR_50_ of 3.1 fold and 5.6 fold, respectively. Mortality rates after 1h exposure to WHO diagnostic dose of deltamethrin were between 84 and 100% in all populations. A significant correlation was between deltamethrin resistance and agriculture activity.

The presence of 88.21% and 94.34% knock-down rates at the end of 1h, KDT_50_ values of 26.02 and 27.71min and KDT_95_ values of 85.36 and 81.18min respectively in the Alanya-Çıplaklı and Alanya-Süleymanlar populations for etofenprox indicate that these populations may develop resistance to etofenprox. The appearance of 94.27% and 96.14% knock-down rates at the end of 1h, the KDT_50_ values of 20.66 and 14.67min and the KDT_95_ values of 69.66 and 45.94min respectively in the Alanya-Çıplaklı and Kumluca-Narenciye populations for lambda-cyhalothrin indicate that these populations may develop resistance to lambda-cyhalothrin.

The knock-down rates, KDT_50_ and KDT_95_ values obtained from susceptibility tests are compatible with the mortality rates at the end of 24h. Mortality rates in the populations with high KDT_50_ and KDT_95_ values with low knock-down rates against etofenprox, deltamethrin and lambda-cyhalothrin were determined to be resistance or possible resistant levels.

### Mortality rates

In this study, the possible resistance or resistance to deltamethrin in all the sampling areas is caused by the widespread use of this active substance against the pests of agriculture and public health for many years. Insecticide resistance in mosquitoes collected from areas where pesticide use is high against agriculture and/or public health pest is higher levels than in areas where pesticide use is less (37–40). Kasap et al. (41) researched resistance to some insecticides of *An. sacharovi* collected in five malaria-endemic provinces of Turkey. According to results of susceptibility tests, Adana, Adıyaman, and Antalya populations were resistant to most insecticides tested, while Aydın and Muğla populations were susceptible to most of the insecticides. Intensive insecticide usage against agricultural pests and mosquitoes has selected resistance in *An. sacharovi* to a number of compounds in these areas. The resistance levels to two types of insecticides of four major vector species (*An. gambiae*, *Cx. quinquefasciatus*, *Aedes* (*Ae.*) *aegypti*, and *Ae. albopictus*) were assessed in Mayotte, a small island in the Indian Ocean (42). Adult bioassays revealed that while field populations of *An. gambiae* and two *Aedes* species were found susceptible to deltamethrin, field population of *Cx. quinquefasciatus* was found stronge resistance to deltamethrin (only 10% mortality after 24h and strong decrease of knock-down effect: RR_50_= 4.2, RR_95_= 4.9). *Culex quinquefasciatus* is the major vector of the Bancroftian filariasis, which has been plaguing Mayotte for many years. Since the 1950s, intense vector control efforts had been carried out by using DDT and followed synthetic pyrethroids against this species. These important selective pressures certainly explain the strong resistance to deltamethrin observed in the *Cx. quinquefasciatus* population. Insecticide resistance status of *Cx. quinquefasciatus* populations from four areas was studied in Benin (43). Adult tests showed high frequency of resistance in mosquitoes to permethrin (ranging from 4% to 24% mortality) and deltamethrin (24% to 48% mortality) in the four research areas. SP have been extensively used in agriculture since 1980s particularly in cotton and vegetable fields in Benin. Moreover, the massive free campaign of bed nets impregnated with permethrin and deltamethrin as the major control strategy against *Wuchereria bancrofti* transmitted by *Cx. quinquefasciatus.* These cases may cause the resistance of *Cx. quinquefasciatus* to SP.

In our study, the highest level of deltamethrin resistance with the lowest mortality rates was detected in mosquitoes collected from the Kumluca-solid waste storage area. This situation is thought to have been caused by the application of residual spraying, thermal and cold fogging by using the SP regularly against the vectors such as mosquitoes, sand flies and house flies by the Antalya Metropolitan Municipality vector control teams in this area. In addition, Kumluca is a district where greenhouse activities and the use of pesticides related to greenhouses is intense in Antalya Province. Therefore, the greenhouse wastes on the solid waste storage area contain intense pesticide residues and the leaking water from these wastes constitutes a breeding site for mosquitoes. Moreover, there are intensively empty packages of various chemicals such as pesticides, fertilizers, and detergents containing residues in the solid waste storage area and agricultural pesticides are used in fields and gardens near to this area.

The resistance levels to deltamethrin of mosquito populations are in agreement with pollution level of the sampling areas and pesticide application frequency in these areas. Alanya-Çıplaklı, Manavgat-Çakış, Döşemealtı-Ilıca, and Kumluca-solid waste storage area populations were resistant to deltamethrin with mortality rates lower than 90%. The collection areas of these four populations are habitats where pollutants such as organic and chemical wastes are concentrated. In addition, vector control applications are regularly carried out by the Antalya Metropolitan Municipal in these four areas and pesticides are used in the fields and fruit gardens near these areas. Pesticides used in agricultural and public health applications are effective for improving resistance of mosquitoes to insecticides. Besides, various anthropogenic or natural xenobiotics in the breeding sites of mosquitoes can also contribute to develop resistance of mosquitoes to insecticides, especially by altering the expression of genes encoding detoxification enzymes and/or cuticular proteins (44). *Aedes aegypti* larvae can be improved resistance to various insecticides in different classes by changes in the expression of genes responsible for detoxification of heavy metal (copper), poly-cyclic aromatic hydrocarbons (fluoranthene and benzo[a]pyrene) or herbicides (glyphosate and atrazine) (45–47). Similarly, the expression of genes encoding cuticular proteins was changed in *Ae. aegypti* larvae exposed to various pollutants and pesticides from different classes (48, 49). Alanya-Süleymanlar, Döşemealtı-Killik, Kemer-Tekirova and Kumluca-Narenciye populations were possible resistant to deltamethrin with mortality rates in the range of 90–97%. The habitats of these populations seem to be cleaner in terms of organic and chemical pollutants when compared to the habitats of the other four populations. In addition, pesticide applications against pests of agricultural and public health are carried out at lower intensity in these sampling areas.

In this study, the permethrin in all sampled locations was the most effective of the four active substances tested, leading to 100% mortality on the mosquito populations. In Turkey, use of permethrin as plant protection product has been terminated by Republic of Turkey Ministry of Food, Agriculture and Livestock since 01 Jan 2011. Therefore, this active substance has not been used against pests of agricultural in Antalya province for the last six years. In addition, permethrin has been used in limited quantities in cold fogging applications against adult mosquitoes by the Antalya Metropolitan Municipality in 2013 and 2015 years. This active substance has not been used against adult mosquitoes in the last two years (50).

As a result of the susceptibility tests, etofenprox was caused 91.54% to 100% mortalities in all populations. While Alanya-Çıplaklı (91.54% mortality) and Alanya-Süleymanlar (91.95% mortality) populations were determined to be possible resistant to etofenprox, the other six populations were found susceptible. Etofenprox is used in low amounts against pests of agriculture and public health in Antalya Province. This case is consistent with the susceptibility to etofenprox of the six tested populations. The possible resistance to etofenprox of the Alanya-Çıplaklı and Alanya-Süleymanlar populations may be due to the local use of this active substance in these sampling areas or development cross-resistance to other insecticides from the used synthetic pyrethroid group in fruit growing (grape, avocado, lemon, orange, medlar, banana etc.) at outdoor area of Alanya District.

Because of tests performed with lambda-cyhalothrin, it was observed mortality in the range of 93.1–100% in all populations. While Alanya-Çıplaklı (93.1% mortality) and Kumluca-Narenciye (96.94% mortality) populations were possible resistant to this active substance, the other six populations were susceptible. Lambda-cyhalothrin is used in low amounts against pests of agriculture and public health in Antalya Province. This case is consistent with the susceptibility to this active substance of the six tested populations. The possible resistance to lambda-cyhalothrin of the Alanya-Çıplaklı and Kumluca-Narenciye populations may be due to the local use of this active substance in these sampling areas or development cross-resistance to other insecticides from the used synthetic pyrethroid group in these sampling areas.

In our literature review, *Cx. pipiens* has developed resistance to SP at different levels in a variety of researches conducted in Turkey and worldwide. *Culex pipiens* populations collected from Aksu district of Antalya were resistant to permethrin (0.75%) and deltamethrin (0.05%) with 78.3% and 75.8% mortality rates, respectively (3). Similarly, *Cx. pipiens* populations collected from Aksu District of Antalya were resistant to permethrin (0.75%) and deltamethrin (0.05%) with 74% and 62% mortality rates, respectively (12). Seasonal dynamics of insecticide resistance were investigated in field populations of *Cx. pipiens* from Çanakkale, Balıkesir, İzmir, Aydın, Muğla, and Denizli provinces in western Turkey (4). In the results of bioassays, all populations showed seasonally different levels of resistance to permethrin and deltamethrin. The resistance status to four insecticides was examined in thirteen populations of *Cx. pipiens* collected from five regions of Greece (39). Adult bioassays showed that while one population was resistant to deltamethrin with 64% mortality rate, three populations were possible resistant with mortalities of 92%, 90% and 87%, and other nine populations were susceptible. Salim-Abadi et al. (51) evaluated the susceptibility status of *Cx. pipiens* collected from the capital city of Tehran, Iran. Field population was resistant to all tested SP (lambda-cyhalothrin 0.05%, deltamethrin 0.05% and cyfluthrin 0.15%). The irritability levels of different groups of insecticides on laboratory strain and field population of *Cx. pipiens* complex were investigated in Tehran, Iran (52). Permethrin (0.75%) and deltamethrin (0.05%) were moderately irritable against both field population and laboratory strain of *Cx. pipiens* complex. While cyfluthrin (0.15%) was moderately irritable for field population, it was hypo-irritable for laboratory strain. Lambda-cyhalothrin (0.05%) was hypo-irritable against both field population and laboratory strain. Whereas etofenprox (0.5%) was hypo-irritable for field population, it was non-irritable for laboratory strain. Ghorbani et al. (53) assessed the susceptibility status to 12 adulticides and two larvicides recommended by WHO of *Cx. pipiens* collected from Sari County in the north of Iran. The susceptibility tests showed that *Cx. pipiens* was resistant to all tested insecticides. Nevertheless, the resistance level was lower to SP compared to the others. The mortality rates after exposure to etofenprox (0.5%), cyfluthrin (0.15%), permethrin (0.75%), deltamethrin (0.05%) and lambda-cyhalothrin (0.05%) were 76.47%, 72.09%, 70.73%, 39.08% and 33.33% respectively. Insecticide resistance may vary among regions, provinces, districts, and even smaller localities within a country. These differences in insecticide resistance include many factors such as the species of mosquito, life stage, physiological status, even the various symbioses or pathogens found in the body of mosquitoes, the climatic characteristics of the study area, altitude, vegetation cover, acreage, socioeconomic structure, agriculture and animal husbandry activities, pesticides used in this area, doses, frequency, and methods of application of pesticides, agricultural chemicals, urban and industrial pollutants (44).

In order to prevent and/or delay to the development of resistance to insecticides in mosquitoes, integrated control programs should be implemented in which chemical use is kept in a minimum level, with emphasis on physical, cultural and biological control methods (15, 54). If chemical use is needed; products with high selective toxicity, low toxicity to non-target organisms, less persistence in the environment and no resistance developed in target organism should be preferred and these products should be used at the doses indicated on the label. In addition, whether mosquitoes have resistance or not should be regularly monitored against the products used in combat (55). In the case of resistance detection, various tests can be performed to determine the mechanism. For this purpose, synergist tests can be used to determine the presence of resistance related to the detoxification enzymes, biochemical enzyme assays to determine the metabolic resistance, and molecular biological tests to determine the target site resistance (20). According to the results obtained from resistance tests, insecticide resistance maps should be prepared. These maps should be used in the selection of insecticide and resistance management (56). Insecticide applications should be made in more limited areas where mosquitoes are heavily infested or mosquito-borne disease risk is high rather than large areas (55). Since larval and adult control must be performed simultaneously to have an effect on mosquito populations, unrelated classes of insecticides with different modes of action should be used for each life stage of mosquitoes (57). It may be beneficial to apply a mosaic approach by using products of different insecticide classes and mode of action in neighboring areas (32, 57). The insecticides with same modes of action should not be used in an area for a long time. Instead, insecticides of different classes with unrelated modes of action should be used in rotation (32, 54, 57, 58). Addition of synergistic substances to products may increase the susceptibility of mosquitoes to insecticides (58). Synergists are compounds that do not have insecticidal activity by themselves. However, when they are mixed with insecticides of a certain class, significantly increase their effect by inhibiting an enzyme that detoxifies the insecticide in the insect body (20, 59). Synergists include piperonyl butoxide (PBO), which inhibits oxidase activity, S.S.S tributlyphosphorotrithioate (DEF), which inhibits esterase activity, ethacrynic acid (EA), diethyl maleate (DM), and chlorfenethol (CF), which inhibit glutathione transferase activity (24). PBO is used as a synergist in insecticide formulations against the public health pests in Turkey. However, there is no standard for synergist ratios to be used in formulations. In addition, the development of more efficient and environment-friendly new compounds with different modes of action, such as herbal, microbial and synthetic origin, as an alternative to the existing insecticides will contribute to preventing or delaying the development of resistance to insecticides in mosquitoes (55, 58, 60).

## Conclusion

In our study, all *Cx*. *pipiens* populations were found to be possible resistant or resistant to deltamethrin. This situation is thought to be due to the widespread use of this active substance in agriculture and public health applications for many years. The resistance levels to deltamethrin of the mosquito populations are related to pesticide application frequency in these areas and the pollution level of the sampling area.

Insecticides are used intensively to control mosquito populations at present. However, mosquitoes develop resistance to almost every kind of insecticides used in combat. This case causes serious concerns. One of the effective ways, in order to prevent and/or delay to the development of resistance to insecticides in mosquitoes, is to minimize usage of insecticide. To achieve this, integrated control programs should be implemented. In addition, the resistance status of mosquito populations against insecticides used in combat should be regularly monitored and insecticide resistance maps should be prepared.

The results of this study will contribute to the planning of the resistance management and selection of insecticides that will be used by the mosquito control agencies and institutions in Antalya, Turkey. However, it needs new studies that will be used for other mosquito species from more localities and different active substances. It is also thought to be useful to conduct further studies to determine resistance mechanism in mosquito populations in areas where resistance is detected.
